# Real-World Experience With Higher-Than-Recommended Doses of Lamivudine in Patients With Varying Degrees of Renal Impairment

**DOI:** 10.1093/ofid/ofy225

**Published:** 2018-09-10

**Authors:** Briann Fischetti, Kushal Shah, David R Taft, Leonard Berkowitz, Anjali Bakshi, Agnes Cha

**Affiliations:** 1 Division of Pharmacy Practice, The Arnold & Marie Schwartz College of Pharmacy and Health Sciences, Long Island University, Brooklyn, New York; 2 Department of Pharmacy, The Brooklyn Hospital Center, Brooklyn, New York; 3 Division of Pharmaceutical Sciences, The Arnold and Marie Schwartz College of Pharmacy and Health Sciences, Long Island University, Brooklyn, New York; 4 Infectious Diseases, The Brooklyn Hospital Center, Brooklyn, New York

**Keywords:** lamivudine, HIV, renal impairment

## Abstract

**Background:**

Although nucleoside reverse transcriptase inhibitors have been associated with lactic acidosis, lamivudine (3TC) has not been reported to have an increased risk with elevated concentrations. Therefore, some recommend that the lowest tablet strength of 3TC be considered in patients with kidney disease to avoid the inconvenience of liquid formations. Our institution avoids dose-adjusting 3TC until creatinine clearance (CrCl) <30 mL/min and uses 100–150-mg tablets daily in hemodialysis. The aim of this study was to describe the use of higher-than-recommended doses of 3TC in a real-world setting.

**Methods:**

Blood samples were collected before and 0.5–1.5 hours after 3TC administration in HIV+ adults. Predose (C_min_) and postdose (C_max_) samples were measured by high-performance liquid chromatography. Physiologically based pharmacokinetic modeling was utilized to simulate areas under the curve (AUCs) and profiles by CrCl. Lactic acid levels and patient-reported adverse events were obtained to monitor for safety, and viral suppression was assessed for efficacy.

**Results:**

Thirty-four patients with varying degrees of renal function were enrolled. Observed 3TC C_max_ values were comparable among CrCl cohorts. Simulated 3TC AUC values in patients with CrCl 30–49, 15–29, and 0–15 mL/min were consistent with historical data, and fold-errors were between 0.5 and 2.0. All lactic acid levels were within normal limits, and no adverse effects were reported.

**Conclusions:**

This study is the first to describe the use of higher-than-recommended doses of 3TC in a real-world setting. 3TC was well tolerated across all levels of renal function. These results can guide providers in their selection of higher 3TC dosing in select patients with renal dysfunction to maximize adherence.

Lamivudine (3TC) is a synthetic nucleoside reverse transcriptase inhibitor with activity against HIV-1 when dosed at 300 mg daily or 150 mg twice daily [1]. Pharmacokinetic studies in asymptomatic HIV-infected patients with normal renal function show that 3TC is rapidly absorbed after oral administration, with maximum serum concentrations (C_max_) reached 0.5 to 1.5 hours after ingestion. 3TC exhibits linear, dose-dependent pharmacokinetics, with C_max_ and area under the curve (AUC) correlating to the strength of the dose administered [2]. Approximately 70% of oral 3TC is eliminated renally, and the package insert recommends dose adjustments for patients with impaired renal function and those on hemodialysis [1, 2]. Negligible amounts of lamivudine are removed by 4-hour hemodialysis or peritoneal dialysis. Supplemental dosing is not required; however, it is recommended to dose after dialysis [1].

3TC package insert renal dose adjustment recommendations were based on the pharmacokinetic results in phase I studies published in 1996 and 2002 [3, 4]. A study conducted by Heald et al. evaluated single-dose pharmacokinetics of 3TC 300 mg in HIV-infected patients with renal impairment [3]. Another study conducted by Bohjanen et al. evaluated steady-state pharmacokinetics of 3TC in HIV-infected patients with end-stage renal disease (ESRD) [4]. These studies found that levels of lamivudine increased with decreasing renal function. Therefore, package insert renal dose adjustments were recommended based on pharmacokinetic modeling.

Lamivudine is generally well tolerated, with common side effects including headache, nausea, and fatigue [1]. However, rare serious adverse effects such as lactic acidosis and hepatomegaly with steatosis have been reported. Although there has been no documented correlation between elevated lamivudine levels and increased risk of lactic acidosis, it may be more likely to occur in females or obese individuals [1]. In the study by Heald et al., although elevated AUC and C_max_ values were observed in patients with varying levels of renal impairment, no adverse events were reported. In addition, Bohjanen et al. found that patients with ESRD on 3TC 150 mg daily had an AUC 5 times greater than those with normal renal function and did not report any adverse events. Investigators concluded that 3TC is a relatively safe drug with a wide therapeutic index.

 In view of the favorable safety profile of 3TC, the lack of data correlating elevated 3TC levels to lactic acidosis, and the inconvenience of using the liquid formulation, some recommend considering the use of the lowest tablet strength of 100 mg or 150 mg daily in patients with advanced kidney disease [5]. Despite the use of higher-than-recommended doses in clinical practice, there is a lack of evidence to support the safety of this practice in the real-world setting. The current practice at our institution is the use of standard 3TC 300 mg daily for patients with a creatinine clearance (CrCl) ≥30 mL/min, to avoid dose-adjusting 3TC until CrCl is <30 mL/min, and the use of 3TC 100–150-mg tablets daily in hemodialysis, which avoids the use of the liquid formulation. A real-world evaluation with an increased sample size would provide a better assessment of the safety of these modified dose adjustments in this patient population.

The objective of this study was to describe the clinical experience of a single US center of patients with renal impairment receiving higher-than-recommended doses of 3TC. The primary end points to evaluate elevated 3TC exposure were mean model-simulated 3TC AUC and mean observed 3TC concentrations predose (C_min_) and 0.5–1.5 hours postdose (C_max_). Secondary end points include HIV RNA, serum lactic acid levels, and patient-reported adverse events.

## METHODS

### Study Population

This cross-sectional study included men and nonpregnant, nonlactating women age 18 years or older with HIV-1 infection receiving 3TC as part of their antiretroviral regimen for at least 3 months. Patients were recruited from a hospital-based outpatient HIV clinic with 2 locations in Brooklyn, New York. Patients were excluded if they were on concurrent nonantiretroviral medications known to cause increased lactic acid levels (metformin, linezolid, theophylline, >3 g of acetaminophen). Patients with a CrCl >30 mL/min were receiving 3TC 300 mg daily. When CrCl was 15–29 mL/min, patients received 3TC 150 mg daily, and when CrCl was <15 mL/min or patients were on hemodialysis, 3TC was dosed at 100–150 mg daily. All patients were receiving tablet formulations of 3TC or in combination products with other antiretrovirals (ARVs). Participants were stratified into 4 groups based on renal function: CrCl ≥50 mL/min, CrCl 30–49 mL/min, CrCl 15–29 mL/min, CrCl <15 mL/min or on hemodialysis. The hospital’s institutional review board approved this study, and all participants completed a written informed consent.

### Study Visits

Participants were instructed to bring their 3TC tablet to their visit. Upon arrival, a blood sample was collected and participants were instructed to take their dose of 3TC. A second blood sample was collected 0.5–1.5 hours after 3TC administration. Plasma concentrations of 3TC in the predose (C_min_) and postdose (C_max_) samples were measured by high-performance liquid chromatography using the method described by Alebouyeh and Amini [6]. Lactic acid levels and serum creatinine (Scr) were also collected, and patient-reported adverse events were documented by study personnel. Scr was used to calculate CrCl using the Cockcroft-Gault equation to stratify the participant into 1 of the 4 renal function groups. Per the institution’s lab, normal lactic acid levels were defined as 0.5–2.2 mmol/L. Any level greater than 2.2 mmol/L was considered elevated. Virologic suppression was defined as <200 copies/mL.

### Data Analysis

The primary end points evaluated were mean model-simulated 3TC AUC (0–24 hours), mean observed 3TC C_min_, and C_max_ concentrations at steady state. Secondary end points included HIV RNA, serum lactic acid levels, and reported adverse events. Part A of the study used physiologically based pharmacokinetic (PBPK) modeling of 3TC systemic exposure carried out using the Simcyp Simulator (version 17). The model was verified against published clinical data based on once-daily dosing of 3TC and fold-error, the ratio of model-predicted and observed data (C_max_, AUC) [7]. Additional models were created for 2 discrete renal failure populations (CrCl 10–40 mL/min and CrCl <10 mL/min). These models were also verified against published data [3, 8]. Model simulations were then used to predict 3TC C_max_ and C_min_ in each study participant, based on gender, age, body weight, and CrCl. The fold-error between simulated and observed concentrations was then calculated. Additionally, 3TC systemic exposure (C_max_, AUC) was simulated in virtual populations (n = 300/group) of patients with varying degrees of renal insufficiency, based on institutional 3TC dosing guidelines determined by CrCl.

Part B of the study describes the clinical experience with using higher-than-recommended doses of 3TC in practice. Patient-reported adverse events were analyzed by a safety monitoring group of infectious diseases physicians to determine the association with 3TC. Descriptive statistics were used to report virologic suppression, elevated lactic acid levels, and patient-reported adverse events.

## RESULTS

There were 34 patients enrolled in the study with varying degrees of renal function. Five patients had normal renal function, defined as a CrCl >50 mL/min, receiving 3TC 300 mg daily. Sixteen patients had a CrCl between 30 and 49 mL/min, receiving 3TC 300 mg daily. Four patients had a CrCl between 15 and 29 mL/min, receiving 3TC 150 mg daily; however, 1 patient was on hemodialysis. There were 10 patients on hemodialysis who were receiving 3TC 100- or 150-mg tablets daily.

### Part A: Pharmacokinetic Analysis

**Figure 1.**
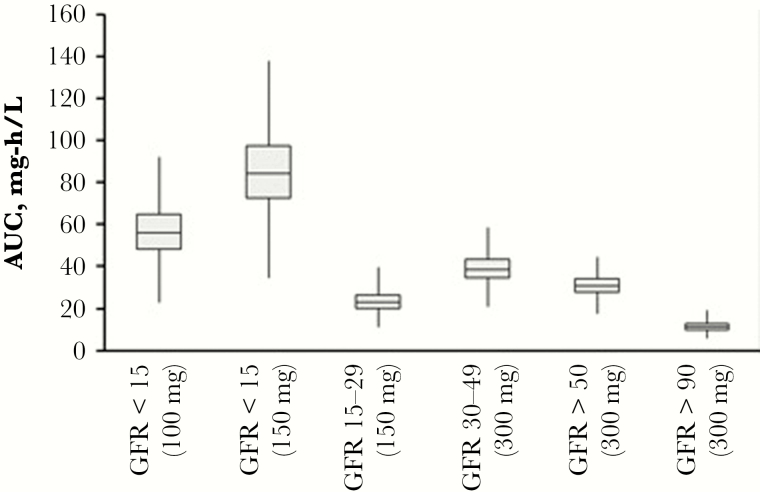
Physiologically based pharmacokinetic model simulated lamivudine area under the curve values based on institutional dosing guidelines. Abbreviations: AUC, area under the curve; GFR, glomerular filtration rate.

Table 1 summarizes the observed and PBPK model-simulated 3TC concentrations in the study participants; the fold-error ranged from 0.49 to 1.73. C_max_ levels were lowest in the normal renal function group (2.93 mg/L) compared with the renally impaired patients: 3.22 mg/L in the CrCl 30–49 mL/min group, 3.48 mg/L in the CrCl 15–29 mL/min group, and 3.21 mg/L in the CrCl <15 mL/min or hemodialysis group. Model-simulated 3TC plasma concentration profiles are presented in Figure 1. Simulations (n = 300/group) were based on institutional dosing guidelines for each CrCl cohort. Steady-state systemic exposure parameters from these simulations are presented in Table 2. C_max_ was lowest in the CrCl 15–29 mL/min group using 150 mg, and AUC was lowest in the CrCl >90 mL/min group using 300 mg, 2.45 mg/L, and 11.2 mg-h/L respectively, with the highest simulated C_max_ of 4.07 mg/L in the CrCl 30–49 mL/min group using 300 mg and the highest AUC of 84.5 mg-h/L in the CrCl <15 mL/min group using 150 mg.

**Table 1.** Observed and PBPK Predicted 3TC Systemic Exposure Across Patient Cohorts

CrCl Group, mL/min	No.	CrCl Observed, mL/min^a^	C_max_, mg/L^b^			C_min_, mg/L^b^		
			Observed	Predicted	FE^c^	Observed	Predicted	FE^c^
>50	5	60.6 (51.8–73.6)	2.93 (1.89)	3.22 (0.59)	1.32 (0.49)	0.60 (0.43)	0.25 (0.16)	0.49 (0.19)
30–49	16	41.1 (31.5–44.3)	3.32 (1.52)	3.12 (0.65)	1.08 (0.43)	0.81 (0.43)	0.55 (0.33)	0.76 (0.49)
15–29	3	21.6 (17.6–25.6)	3.48 (1.28)	3.58 (0.26)	1.73 (0.90)	1.34 (0.73)	1.81 (1.19)	1.69 (1.63)
<15 or hemodialysis	10	9.1 (3.4–17.5)	3.21 (1.55)	3.62 (1.40)	1.30 (0.67)	1.09 (0.98)	1.06 (0.74)	1.45 (1.43)

Abbreviations: 3TC, lamivudine; C_max_, maximum serum concentration; C_min_, minimum serum concentration; CrCl, creatinine clearance; FE, fold-error; PBPK, physiologically based pharmacokinetic.

^a^Data presented as median (range).

^b^Data presented as mean (SD).

^c^Fold-error, ratio of predicted:observed values.

**Table 2.** PBPK Model Simulated 3TC Systemic Exposure Based on Institutional Dosing Guidelines^a^

CrCl Group, mL/min	Daily Dose, mg	Steady State C_max_, mg/L	T_max_, h	Steady State AUC(0-τ), mg-h/L
> 90	300	2.45 (0.44)	1.08 (0.17)	11.2 (2.09)
>50	300	3.70 (0.67)	2.08 (0.25)	30.8 (4.80)
30–49	300	4.07 (0.70)	2.30 (0.29)	39.0 (6.37)
15–29	150	2.19 (0.38)	2.45 (0.34)	23.2 (4.23)
<15	150	3.11 (0.60)	4.66 (0.90)	84.5 (18.2)
	100	3.11 (0.60)	3.36 (0.33)	56.3 (12.1)

Abbreviations: 3TC, lamivudine; AUC, area under the curve; C_max_, maximum serum concentration; C_min_, minimum serum concentration; CrCl, creatinine clearance; PBPK, physiologically based pharmacokinetic; T_max_, time to reach C_max_.

^a^Data are presented as mean (SD) from simulations based on 300 virtual subjects.

### Part B: Clinical Experience

The majority of patients were nonwhite (88%), and all were virologically suppressed on antiretroviral therapy (ART; 100%) with HIV RNA <200 copies/mL. All patients had undetectable HIV RNA <20 copies/mL, with the exception of 2 patients, who had virologic blips of 34 and 140 copies/mL at the time of data collection. Baseline characteristics and ART regimens are listed in Table 3. The range of duration of treatment with 3TC varied widely, with 2 patients being on 3TC for 3–6 months and the remainder reporting 2–5 years or >5 years. None of the study subjects had elevated lactic acid levels, and there were 0 patient-reported adverse effects.

**Table 3.** Patient Characteristics

Characteristic	n = 34
Mean age (range), y	63 (37–84)
Mean weight (range), kg	76 (38–127)
Race	
Black, No. (%)	23 (68)
Hispanic, No. (%)	7 (20)
White, No. (%)	4 (12)
Female sex, No. (%)	17 (50)
CrCl >50 mL/min, No. (%)	5 (15)
CrCl 30–49 mL/min, No. (%)	16 (47)
CrCl 15–29 mL/min, No. (%)	4 (12)
CrCl <15 mL/min or HD, No. (%)	9 (26)
AIDS, No. (%)	12 (35)
Hypertension, No. (%)	27 (79)
Diabetes, No. (%)	10 (29)
Hepatitis C virus, No. (%)	11 (32)
Cardiovascular disease, No. (%)	13 (38)
Psychiatric disorders, No. (%)	9 (26)
Smoker, No. (%)	13 (38)
HIV RNA <200 copies/mL, No. (%)	34 (100)
Mean CD4 count (range), cells/mm^3^	639 (145–1552)
Statin, No. (%)	23 (68)
Antihypertensive, No. (%)	26 (76)
ART regimens, No. (%)	
3TC 300 mg + NRTI + INSTI	12 (35)
3TC 300 mg + NRTI + NNRTI	4 (12)
3TC 300 mg + NRTI + NNRTI + INSTI	4 (12)
3TC 300 mg + NRTI + PI	2 (6)
3TC 300 mg + NRTI + INSTI + PI	1 (3)
3TC 150 mg + NRTI + INSTI	4 (12)
3TC 150 mg + NRTI + PI	2 (6)
3TC 150 mg + INSTI + PI	1 (3)
3TC 150 mg + NRTI + INSTI + PI	1 (3)
3TC 100 mg + NRTI + INSTI	2 (6)
3TC 100 mg + INSTI + PI	1 (3)

Abbreviations: 3TC, lamivudine; ART, antiretroviral therapy; HD, hemodialysis; INSTI, integrase strand transfer inhibitor; NRTI, nucleoside reverse transcriptase inhibitor; NNRTI, non-nucleoside reverse transcriptase inhibitor; PI, protease inhibitor.

## DISCUSSION

This study is the first to describe the use of higher-than- recommended 3TC doses in a real-world setting. Although there is pharmacokinetic literature published on 3TC use in renal impairment, it has been limited to hemodialysis pharmacokinetics and single-dose studies with small sample sizes [2–4]. The study conducted by Heald et al. included 6 patients with normal renal function, defined as CrCl ≥60 mL/min, 4 patients with moderately impaired renal function, defined as CrCl 10–40 mL/min, and 6 patients with severely impaired renal function, defined as a CrCl <10 mL/min [3]. Three of the 6 patients with severely impaired renal function were on hemodialysis (HD), and the other 3 were on peritoneal dialysis [3]. Bohjanen et al. studied only 11 patients with ESRD, 9 of whom were on chronic HD [4]. This study is the first to report on steady-state 3TC concentrations in patients with varying levels of renal impairment receiving higher-than-recommended doses.

### Part A: Pharmacokinetic Analysis

Observed 3TC C_max_ levels were comparable among CrCl cohorts, adjusting for the dose given. As expected, C_min_ was elevated in renally impaired patients, due to reduced 3TC clearance and longer t_1/2_. PBPK model simulations captured the data well, with fold-errors between 0.5 and 2.0.

Additional PBPK model simulations were performed in patient populations and doses matching institutional guidelines (Table 2). These results are consistent with published data. Heald et al. reported a median AUC (0-infinity) of 11.3 mg-h/L following single-dose administration to subjects with normal renal function (CrCl >60 mL/min) [3]. Although PBPK model simulations in a virtual population with CrCl >50 resulted in 3-fold higher exposure (simulated AUC, 30.8 mg-h/L), the normal renal function subjects (n = 6) in the Heald et al. study had mean CrCl values of 110 mL/min compared with mean CrCl values of 60.1 mL/min in this study group of CrCl >50 mL/min. When model simulations are restricted to subjects with CrCl >90 mL/min, the model-estimated AUC (11.2 mg-h/L) shown in Table 2 compares favorably with Heald et al. and Bouazza et al. [3, 9]. Likewise, when adjusted for dose, simulated 3TC AUC levels in patients with renal insufficiency (CrCl 30–49, 15–29, and 0–15 mL/min) are consistent with observed data in patients with moderate and severe renal impairment from the Heald et al. study (45.7 and 146.5 mg-h/L) [3]. Figure 1 depicts simulated AUC values across all renal function groups.

**Figure 2.**
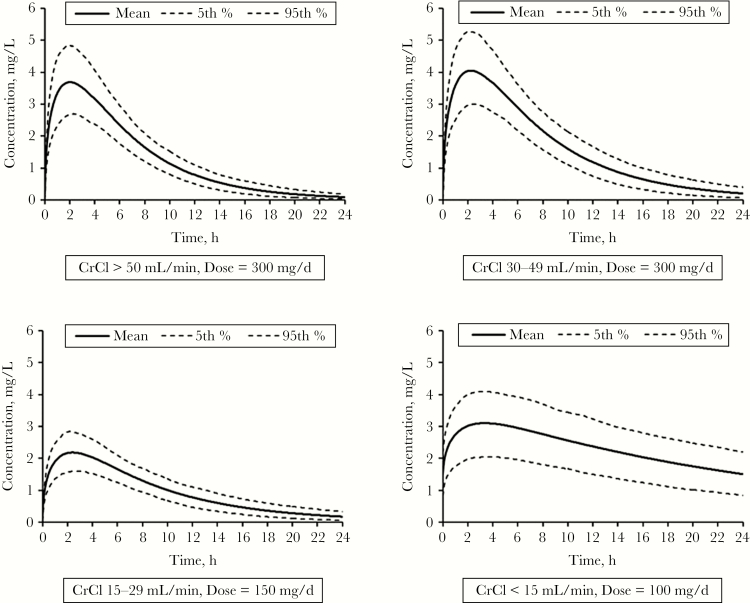
Physiologically based pharmacokinetic model predicted steady state lamivudine plasma concentrations vs time profiles in virtual populations with varying degrees of renal impairment following once daily dosing. Abbreviation: CrCl, Creatinine Clearance.

Overall, the results of this investigation demonstrate that 3TC exposure was comparable among treatment cohorts when using higher-than-recommended doses. In patients with moderate to severe renal insufficiency, the plasma concentration-time profile has a flatter shape with a longer T_max_, due to a longer t_1/2_ (Figure 2). Therefore, while C_min_ levels are elevated in these subjects, C_max_ levels are not.

Due to the nature of the real-world setting, the study was limited to only 2 serum concentrations, and the sampling scheme for C_max_ (sample 0.5–1.5 hours after dosing,) and C_min_ (unknown timing relative to previous dose) likely contributed to the fold-error in PBPK model predictions. There was also a lack of documentation of food intake, which may affect 3TC C_max_ levels as model simulations were done in the fasted state. Timing of plasma concentrations in relation to last dialysis session was not documented. In addition, Simcyp modeling for virtual populations was not able to account for hemodialysis removal of 3TC, which may be a factor in the elevated steady-state AUC in patients with CrCl <15 mL/min, displayed in Figure 1. However, as HD removes negligible amounts of 3TC, this was not likely to significantly affect the results.

Although a fairly small number of patients were enrolled, this represents a larger sample size (n = 34) in comparison with the pharmacokinetic studies used to determine renal dose adjustments in the manufacturer package insert (n = 16). In addition, PBPK modeling in Simcyp was carried out in virtual populations of 300 subjects, and the results remained consistent with the institutional dosing guidelines for 3TC in select patients.

### Part B: Clinical Experience

All patients enrolled in this study had normal lactic acid levels, and there were 0 patient-reported adverse effects. Considering that all patients had been on their respective doses of 3TC for at least 3 months, with the majority of patients reporting use of 3TC for >5 years, higher-than-recommended doses of 3TC were well tolerated in this select group of patients. However, it is important to note that patients who may not have tolerated higher-than-recommended doses may have discontinued 3TC before enrollment and therefore would have been excluded from this study. Due to the cross-sectional design, investigators were unable to determine how many patients discontinued 3TC previously due to possible adverse effects as their renal impairment progressed. Additional limitations include the single-center location and the subjective assessment of self-reported adverse effects.

Our study provides experience in using 3TC 300 mg daily in select patients with a CrCl of 30–49 mL/min, and 100–150 mg daily in patients with a CrCl <30 mL/min or on hemodialysis. This study describes a small cohort of patients who had been on higher-than-recommended doses for at least 3 months before enrollment in this study. There may have been patients who were unable to tolerate 3TC and experienced adverse effects that were discontinued before this evaluation. In patients who have declining renal function and have difficulty tolerating 3TC, doses should be reduced to reflect package insert recommendations. However, for patients able to tolerate our modified dosing recommendations, this would allow for a significant impact on patient adherence as coformulated 3TC products would not need to be separated into individual components to adjust 3TC dosing until CrCl falls below 30 mL/min. In addition, patients with severe renal impairment or on hemodialysis would be able to use 3TC 100-mg or 150-mg tablets instead of using a liquid formulation, which would allow for easier administration.

## CONCLUSIONS

Doses of 3TC 300 mg in patients with a CrCl >30 mL/min and 100–150 mg daily in patients with a CrCl <30 mL/min or receiving hemodialysis were well tolerated in our patient population. PBPK modeling demonstrated that simulated 3TC C_max_ and AUC values were comparable among patient cohorts, and there were no observed elevated lactic acid levels or patient-reported adverse effects in the study. All patients were virologically suppressed. Conducting a large clinical trial would be necessary to establish the true safety and efficacy of using higher-than-recommended doses of 3TC in renally impaired patients. However, the results from this real-world cohort may help guide providers in their selection of 3TC dosing in select patients with renal impairment who are tolerating 3TC, to optimize their adherence by continuing to use coformulated products and avoid the use of liquid formulations.
